# General ecological information supports engagement with affordances for ‘higher’ cognition

**DOI:** 10.1007/s11229-018-1716-9

**Published:** 2018-02-12

**Authors:** Jelle Bruineberg, Anthony Chemero, Erik Rietveld

**Affiliations:** 1 Institute for Logic, Language and Computation (ILLC), University of Amsterdam, Oude Turfmarkt 141, 1012 GC Amsterdam, Netherlands; 2 Center for Cognition, Action and Perception, University of Cincinnati, Cincinnati, OH USA; 3 Department of Philosophy, University of Cincinnati, Cincinnati, OH USA; 4 AMC/ILLC/Amsterdam Brain and Cognition, University of Amsterdam, Amsterdam, Netherlands

**Keywords:** Affordances, Ecological psychology, Information, Higher cognition, Imagination, Intentionality

## Abstract

In this paper, we address the question of how an agent can guide its behavior with respect to aspects of the sociomaterial environment that are not sensorily present. A simple example is how an animal can relate to a food source while only sensing a pheromone, or how an agent can relate to beer, while only the refrigerator is directly sensorily present. Certain cases in which something is absent have been characterized by others as requiring ‘higher’ cognition. An example of this is how during the design process architects can let themselves be guided by the future behavior of visitors to an exhibit they are planning. The main question is what the sociomaterial environment and the skilled agent are like, such that they can relate to each other in these ways. We argue that this requires an account of the regularities in the environment. Introducing the notion of *general ecological information*, we will give an account of these regularities in terms of constraints, information and the form of life or ecological niche. In the first part of the paper, we will introduce the skilled intentionality framework as conceptualizing a special case of an animal’s informational coupling with the environment namely skilled action. We will show how skilled agents can pick up on the regularities in the environment and let their behavior be guided by the practices in the form of life. This conceptual framework is important for radical embodied and enactive cognitive science, because it allows these increasingly influential paradigms to extend their reach to forms of ‘higher’ cognition such as long-term planning and imagination.

## Introduction

The main theoretical concepts of ecological psychology are *affordance* and *ecological information * (Gibson 1979/2015). Affordances are possibilities for action provided by the environment. Ecological information is the set of structures and regularities in the environment that allow an animal to engage with affordances. An important open question is how far the applicability of the affordance-framework reaches. Important theoretical developments have been made concerning the concept of affordances in order for it to be applicable to more socio-cultural aspects of human and animal activities (Chemero 2003a, 2009; Heft 2001; Reed 1996; Rietveld and Kiverstein 2014; Stoffregen 2003). In this paper, we investigate how we should conceptualize the corresponding notion of ecological information.

For some of Gibson’s later followers (e.g. Turvey et al. 1981), information is to be understood in terms of lawful relationships between structures in the environment and patterns in light, vibrations, and the like. On this account, affordances are perceivable in virtue of the availability of structures in the ambient array (say, a light pattern) that lawfully specify the presence of affordances. At the same time, Gibson himself states that understanding the affordances of things and other humans “comprises the whole realm of social significance for human beings” (1979/2015, p. 121). That is to say, ecological psychology is supposed to be able to deal with the full range of human social activities, not just with simple sensorimotor coordination.

There is a tension between this emphasis on lawful specification and the claim that affordances can be applied to the whole domain of human interactions. Some authors take a narrow approach, limiting affordances exclusively to those action possibilities that are lawfully specified by information (e.g. Turvey et al. 1981; Runeson and Frykholm 1983; Golonka 2015; Golonka and Wilson 2016), while others understand affordances more broadly, relating them to the full range of our human form of life, structured by conventions, customs, socio-cultural practices, or other regularities (Costall 1999; Heft 2001; Rietveld and Kiverstein 2014; Bruineberg et al. 2016; Van Dijk and Rietveld 2017). These two strands of research use different definitions of affordances (see Chemero 2003a; Rietveld and Kiverstein 2014) and require a different account of information.^1^ In this paper we focus on the human form of life in all its richness and therefore take the broader approach. We are concerned with the informational structures that are able to support the whole realm of skilled human activities.^2^

We will argue that what we call ‘the human form of life’ is replete with (partially non-lawful) regularities that support our interactions with the environment. These regularities do not just support visually guided actions, but also activities that are traditionally seen as ‘higher cognition’ such as imagination and long-term planning.

In the first section of this paper, we present the Skilled Intentionality Framework (SIF) (Rietveld and Kiverstein 2014; Bruineberg and Rietveld 2014; Van Dijk and Rietveld 2017; Rietveld, Denys and van Westen forthcoming), a philosophical, ecological-enactive approach to understand the situated and affective embodied mind. SIF follows the guidelines of radical embodied cognitive science (Chemero 2009) to account for cognition, action, and perception in terms of dynamical systems theory and organism-environment coupling, and without invoking mental representations. In the second section of the paper we present different ways to analyse the structure of the human form of life and identify the notion of a constraint as a useful term in which to understand both lawful and non-lawful regularities. In the third section of the paper, we analyse how regularities in the form of life can support forms of cognition that are typically seen as forms of ‘higher cognition’.

## The skilled intentionality framework

The basic phenomenon that we are interested in, in this paper, is how a skilled animal can coordinate its behavior with a complex and dynamical environment. By this we mean that the animal is sensorily coupled to only a locally present aspect of its environment and still is able to coordinate its behavior with respect to distal aspects of the ecological niche. This requires an understanding of the relations between aspects of the environment such that a skilled agent can, based on its skills and learning history, coordinate adequately with it. Before we can move to the environmental regularities presupposed in this coupling, we first present the animal-environment coupling that forms the background of this paper. We will look at this organism-environment coupling in terms of three complementary perspectives: normativity, intentionality, and dynamics.

First, the animal’s coupling with the regularities in the environment is about appropriate behavior: for example there is something distinctively inadequate about waiting for a train in the middle of a meadow. Thanks to an extended process of the education of attention, mature actors have been introduced into their ecological niche by more experienced practitioners and are typically able to act in appropriate ways in concrete situations they encounter; they have become skilled (Rietveld 2008). Second, we follow the phenomenological tradition of Heidegger and Merleau-Ponty in realizing that our skills give us a distinct mode of intentional access to the world. The skilled animal perceives its environment in terms of the action possibilities that *matter* to it. Skilled access to relevant aspects of the world is the norm in cognition, not an exception (Noë 2012; Rietveld et al. Forthcoming; Ingold 2011). Third, from a dynamicist perspective we can understand skilled agency in terms of the dynamics of animal-environment systems, including the neurodynamics embedded or “nested” in the dynamics of the entire brain-body-environment system (Chiel and Beer 1997; Byrge et al. 2014).

The skilled intentionality framework (SIF) attempts to integrate these three perspectives on an agent’s interactions with the environment in one framework. Skilled intentionality is defined as the selective engagement with multiple affordances simultaneously. Some of the affordances we encounter in a specific situation go unnoticed, others solicit an action. Work by Rietveld (2008, 2012) and by Withagen et al. (2012, 2017) has shown that ecological psychology needs to distinguish between “affordances” and “invitations” or “solicitations”.

We^3^ have defined affordances at the level of the form of life as a whole (the ecological niche), namely as relations between aspects of the sociomaterial environment and abilities available in form of life (Rietveld and Kiverstein 2014). For understanding the engagement of a particular individual agent we need to look at the following two relata: the agent’s ability and the aspect of the sociomaterial environment (Chemero 2003b, 2009). The notion of affordances characterizes what is *possible* for agents to do. Solicitations, on the other hand, characterizes what stands out for the individual agent as *relevant* to do in a particular situation. While sitting at a desk writing a journal article, there are lots of things an academic could do: watering the plants, rearranging books, going home early. However, in the current context the solicitations are, if all goes well, limited to the keyboard, the screen, a cup of coffee, and a small pile of papers. These solicitations or attracting affordances involve an experienced tension of something that stands out to be done (Merleau-Ponty 1945/2002; Dreyfus and Kelly 2007). The theory of affordances therefore needs a theory of agency or skilled intentionality, of how an individual agent can be selectively open to only the relevant affordances in a given situation.

In Bruineberg and Rietveld (2014), we have developed a largely Gibsonian and Merleau-Pontyian account of skilled intentionality, which also complements the account of information that we will develop in this article. We have characterized skilled intentionality as the organism’s tendency towards an optimal grip on a whole field of solicitations. In order to tend towards grip, we have to be *selectively open* to only the relevant affordances. We have argued that selective openness to affordances is constituted by the skilled animal’s anticipatory dynamics, understood in terms of self-organizing states of action readiness, which are forms of action preparation (see also Bruineberg et al. 2016; Rietveld et al. Forthcoming; Frijda 2007).

A key fact about skilled action is that of all the things an academic *could* do in her office, the vast majority of actions would be inappropriate, weird or forbidden. Her selective openness to affordances is appropriate with respect to a socio-cultural practice or *form of life* (Rietveld and Kiverstein 2014; Wittgenstein 1953, 1993): The form of life of a kind of animal consists of behavioral patterns, i.e., relatively stable and regular ways of doing things. For humans, these regular patterns in people’s activities are manifest in normative behaviors, customs and conventions in communities. That is to say, human beings share not only biology, but also embedding in sociocultural *practices*. We share more or less stable ways of living with others (cf. ‘*feste Lebensformen*,’; Wittgenstein 1993, p. 397).” (Rietveld and Kiverstein 2014). The richness of affordances here and now for the academic are available because she partakes in the form of life of humans and socio-cultural practices of academics, Macbook users etc. At the same time, her participation in these practices also limits which affordances can stand out as relevant in a given context. When she enters the university library, she adapts her gait, she will whisper rather than talk out loud and will switch her phone to silence-mode. As such her activities also contribute to the regular way of going on; to maintaining the conventions and socio-cultural practice of the library.

On the one hand, SIF attempts to provide an accurate conceptualization of skilled action; on the other hand, the question is how such episodes of skilled action are possible. Skilled intentionality, i.e. coordinating with multiple relevant affordances simultaneously, requires the animal to be sensitive to and adapt to regularities and structures that are there in the environment. In the rest of this paper, we explore how this selective sensitivity is possible, and how to conceptualize the regularities that are there in the environment. We will see that these regularities involve the whole form of life. This brings us, in the last section, a step closer to how skilled intentionality might be applicable to what is traditionally understood as ‘higher cognition’.

## The structure of the environment

The central question of this section is the following: what is the structure of the world at the ecological scale such that animals can couple with it in ways that result in organized, adaptive and creative behavior? An observation made by a long list of thinkers in philosophy, psychology and biology, is that the world at the ecological scale is quite unlike the world as typically studied by physics (von Uexkull 1934/1992; Merleau-Ponty 2003; Jonas 1966/2001; Gibson 1979/2015; Ingold 2000, 2011; Thompson 2007). One reason for this is the environment of the animal is not the equilibrium world of Newtonian physics and classical thermodynamics, but rather an environment full with optical, acoustic, vibrational and pheromonal fluxes and patterns, partly generated by the movement and the presence of the animal itself.

In *The Ecological Approach to Visual Perception *(1979/2015), Gibson devotes nearly half the book on the structure of the environment such that animals can directly perceive it. For Gibson, the main concept involved in understanding the direct coupling of the organism with the affordances in the environment is that of *ecological information*. Ecological information is traditionally understood as a relation between energy in the medium (in the form of light, vibrations etc.), and the substances and surfaces of the environment. Structured light from, for instance, the sun bounces of the surfaces and structures in the environment so that at each point of observation, the light carries information about the structures it has bounced of off. Gibson goes to great pains to show that the information in the environment is rich enough to be able to specify the structures and surfaces in the environment and the perceiver’s relationship to these structures. For Gibson:

[t]he central question for the theory of affordances is not whether they exist and are real, but whether information is available in the ambient light for perceiving them (Gibson 1979/2015, p. 140)

For example, Sedgewick (1973) points out that the horizon cuts across objects at a height that is equal to the height of the point of observation. That is to say, whenever light is reflected to some point of observation from the horizon, and from some object between that point and the horizon, then the light at that point of observation can be used to perceive affordances related to the relative height of that object and the observer, such as the ‘reachability’ and the ‘pass-under-ability’ of an object for an observer.

Some of Gibson’s most influential later followers (e.g. Turvey et al. 1981) focused on information provided by lawful relationships between structures in the environment and patterns in light, vibrations, and the like. However, importantly, lawful relations are not sufficient to account for the richness and diversity of the affordances available in the human form of life (Rietveld and Kiverstein 2014). The overwhelming majority of affordances in human social relations are not lawfully specified by the energy in the environment, but are determined in part by socio-cultural practices, such as conventions and customs, or other regularities. For instance, a colleague might make coffee every morning and put it in a thermos. The thermos normally affords pouring coffee, despite the fact that occasionally the colleague is ill, and the thermos is empty, or contains the cold coffee of the day before. Although these regularities are not strictly law-like, since there can be exceptions (like in this case of illness), they do form the basis for the majority of our everyday skillful engagement with the environment in the human form of life. We will see that this is crucial.

As mentioned above, for Gibson, understanding the affordances of things and other humans “comprises the whole realm of social significance for human beings” (Gibson 1979/2015, p. 121). That is to say, ecological psychology is supposed to be able to deal with the full range of human social activities, not just with simple sensorimotor coordination. In the human form of life, these activities include creativity, long-term planning and imagination. Although the law-based notion of information is able to couple the light hitting our retina with the substances and surfaces in the environment, it is ill-equipped to couple us to the intricate patterns in human sociomaterial practices. Still, human living systems rely on these latter patterns of activities or regularities for most of the distinctively human things that they do. For instance, the supermarket at the corner correlates reliably enough with the presence of bananas to enable banana-oriented behavior, even though every now and then the bananas happen to be out of stock there.

*Lawful and general ecological information*

In this paper, we will therefore distinguish between *lawful ecological information *and *general ecological information*. The former notion, especially as used by Turvey et al. (1981), pertains to a lawful regularity in the ecological niche between structure at a point *p* in a medium and an aspect of the environment at point *q* such that there is a 1:1 specifying relationship between *p* and *q*. When an animal is at *p*, it can be perceptually coupled to the affordances of *q*. We will call this restricted notion of ecological information *lawful ecological information*. In order to account for the sociomaterial character of the environment in the human ecological niche, we argue that a more general account of *ecological information *is required (henceforth *general ecological information*).

*General ecological information* is any regularity in the ecological niche between aspects of the environment, *x* and *y*, such that the occurrence of aspect *x* makes the occurrence of aspect *y* likely. Because of the regular relation between the aspects of the (sociomaterial) environment *x* and *y*,  general ecological information allows an animal to couple to a distal (i.e. not sensorily present) aspect of the sociomaterial environment.

General ecological information pertains to the ways in which aspects of the environment tend to occur together, like smoke and fire, an object and a shadow, or a pub and beer. Of course, an individual’s adaptive behavior requires that the animal is able to couple to the relevant aspect of the environment. This depends on the abilities of an agent and the properties of the aspect of the environment i.e. a blind animal can’t couple to a pattern in the light, and a human can only couple to the message on his phone by coupling to the light that shines off of it.

General ecological information is not limited to aspects of the environment that are sensorily present: something (say a bird of prey, aspect *y*) does not need to be sensorily present to get me ready to act on its affordances, because its shadow (the shadow of the bird, environmental aspect *x*) can reliably inform me about the presence of aspect *y*, even though in exceptional cases the shadow (aspect *x*) might be caused by a different aspect of the environment than aspect y (say for example aspect *z*, a kite). This example of the bird and its shadow also shows that the case of such *general ecological information*—due to the regularities in our ecological niche—is such that an aspect of the environment constrains (but does not necessarily specify lawfully) another aspect of the environment. *Lawful ecological information* also depends on regularities in our ecological niche and can best be seen as a special case of *general ecological information*. *Lawful ecological information* is a special case, or subclass, of *general ecological information *understood from the perspective of regularities: all regularities between aspects of the environment constitute *general ecological information*, but only regularities that determine (rather than constrain) the state of another aspect of the environment constitute *lawful ecological information.*^4^

Our notion of ecological information^5^ (both lawful and general) pertains to regular relations between aspects of the environment (patterns, events, substances and surfaces etc.). This notion of ecological information is minimal, cheap and ubiquitous, in the sense that it is present whenever there are regularities. It is informational in the sense that regularities that hold between aspects of the environment allow animals *to be informed *about one aspect of the environment by the presence of another aspect. To avoid misunderstandings, we stress that this sort of information does not imply meaning or “aboutness” encoded in a signal.^6^ Information is nothing over and above these regularities between aspects of the environment.

*General ecological information and constraint*

Following situation semantics (Barwise and Perry 1981, 1983) we will use the term “constraint” for the regularities between situations that reduce possibilities. Whenever there are constraints between types, there is information between tokens. An example that (Chemero 2009) provides is the situation in which there is an unopened beer can on a table in a brightly lit room. Light from the source will reflect off the beer can and off the other surfaces in the environment. At each point in the room in which there is an uninterrupted path from the beer can, there will be light that has reflected of the beer can and is structured in a peculiar way. Due to the natural laws governing the reflection of light off surfaces and textures, the light at any such point in the room will be structured in a very particular way. In this case, there is a lawful constraint connecting the light-structure of type *A* to the beer-can presence of type *B*. In virtue of this constraint, the light structure at point *p* contains information about token beer-can-presence *b* (of type *B*) at some other point $p'$. Furthermore, and crucially for understanding our proposal, in our socio-cultural practices it is generally the case that unopened beer cans contain beer. Because of these conventional constraints governing cans and their contents, beer-can-presence *b* being of type *B* carries information about beer-presence *c* of type *C*. That is to say, because of the constraints and regularities, both physical and conventional, the light (ambient array) at some point in the room can carry information about the availability of beer.

Importantly, this sociomaterial regularity allows for exceptions: a mistake at the brewery might cause the can to be filled with water rather than beer. In the most basic cases, it is the *use of information* rather than *information* itself that is normatively evaluable.^7^ In other words, there is nothing wrong (nor right!) about the light bouncing off a beer-can when it is accidentally filled with water. The light-structure of type *A* specifies beer-can presence no matter what: it carries information about beer-can presence without being able to be right or wrong. It is someone’s *use* of information (by for example drinking the beer or stating that “this can contains beer”) that is normatively evaluable. In the case of human made artefacts, on the other hand, both the information itself and its use are normatively evaluable. If, by some other mistake in the factory, soda-cans end up with beer labels, the label *misrepresents* the contents of the can (even though the light bouncing of the can still specifies a beer-can). In summary, the use of information is always normative; human artifacts that contain information are subject to norms; energy arrays contain information but are not subject to norms.

Information pertains to the constraints that exist in the (sociomaterial) environment. These constraints can be necessary, such as the principle of non-contradiction, nomological or lawful, such as the laws of optics, or conventional, such as a thermos containing coffee or a supermarket selling bananas. While ecological psychology has typically focussed on finding lawful constraints, we are interested in the combination of lawful and conventional constrains that allow us to understand the relation between affordances, ecological information and the human form of life in all its complexity. The information induced by conventional constraints differs from the lawful constraint in that it is not exception-free. The light structure of type fridge-presence does not infallibly specify the presence of beer in the fridge, but can still carry information about beer-presence if there is a constraint between the types of fridge-presence and beer-presence.

A related notion of constraint-based ecological information, also departing from Barwise and Perry (1983), is proposed by Sverker Runeson (1988, 1989). However, Runeson uses conventional constraints to argue that information is specific even though purely lawful constraints do not specify the layout of the environment.^8^ In this paper, we are not committed to the claim that perception needs to be specific in order to guide behavior.

Although constraints and regularities serve different purposes, they are not necessarily different things. To give an example: the convention of driving on the right puts constraints on the possible layout of intersections (e.g. the placing of road signs) and the location of the steering wheel in an automobile. The fact that this convention holds across continental Europe is a regularity that allows the exercise of the skill of driving, which was learned at one particular location, all over the continent.

Most of a skilled agent’s interactions with the environment are far less explicit and articulable. Barwise and Perry (1981) provide the example that what makes someone a skilled basketball player is “her extensive *implicit* knowledge of the constraints that affect her, the ball, and the other things on the court” (p. 98). Although, in order to clearly lay out our theory of information, we focus in this paper on relatively common sense and straightforward examples, such as beer cans and fridges, we recognize that ecological psychology is at its best when uncovering non-trivial regularities that agents use to coordinate their behavior, ranging from perceptual variables such as time-to-impact (Lee and Reddish 1981) to multi-modal informational structures enacted by co-performers that constrain the behavior of interacting jazz musicians (Walton et al. 2015).

*Constraints, information and form of life*

Now let us try to apply the above account of information to the notion of a form of life. As we have seen before, the form of life of a kind of animal consists of patterns in its behavior, i.e., relatively stable and regular ways of doing things. The notion of a “form of life” allows us to capture the variety of socio-cultural practices within the human way of life. In the human form of life, the affordances available are related to the whole spectrum of abilities available in our human socio-cultural practices.

Wittgenstein shows the dependency of the human form of life on regularities as well as the interwovenness of the material and the socio-cultural aspects of our environment with an example of a familiar practice:

[...] if things were quite different from what they actually are - if there were for instance no characteristic expressions of pain, of fear, of joy; if rule became exception and exception rule; or if both became phenomena of roughly equal frequency – this would make our normal language-games lose their point. – The procedure of putting a lump of cheese on a balance and fixing the price by the turn of the scale would lose its point if it frequently happened for such lumps suddenly grew or shrank for no obvious reason. (Wittgenstein 1953, p. 48, PI 142).

In our human environment, the conventional practices (say, of weighing cheese in order to price it), can only exist in virtue of lawful stabilities in nature (most objects having a relatively stable weight over time).

The interwovenness of the material and the socio-cultural aspects of the form of life allows for a multiplicity of such dependencies. There is a regularity between the location of the steering wheel in a car, the material layout of roads and the socio-cultural norm of driving on the right. The fact that only driving left and right exist as stable norms might further be due to the fact that human and automobile locomotion takes place on surfaces and in gravitational circumstances that rule out passing on top or below. For scientific purposes, it might be interesting to investigate the historical or causal priority of some of these constraints; right-hand traffic existed before cars existed so the former constrained the latter and not vice versa. One might very well argue that the constancy of the gravitational force and the properties of cheese constrain and make possible the practice of weighing cheese.

But from the perspective of a participant in the practice, what exactly brings these regularities about is irrelevant. All that is required is a sensitivity to the regularities that are there in the form of life such that, when for example approaching an intersection, the affordances that show up as relevant are in line with the regular ways in which things are done in the particular practice. To use another example of Barwise and Perry (1981), a skilled veterinarian does not need to possess a theory of how X-rays work in order to perceive that the dog’s leg is broken. For the veterinarian, it suffices to be sensitive to the constraint the state of the dog’s leg puts on the pattern on the X-ray, even though this constraint itself is the result of a complex interplay between the dog’s leg, electrons and the detector. In effect, given the physical, technological, and conventional practices in which the veterinarian engages, and her skills acquired in these practices, she can see through the X-ray to the dog’s leg in the same way that she can see through the window to the trees outside. In both cases, there is information available to enable perception.

Many of our everyday activities are founded on conventional constraints. For example, at home one can look out of a window and see the roof of an arriving tram. Based on familiarity with the sociomaterial practice of tram 3 in Amsterdam, one can apprehend that this is the end point of the tram and that normally it will stop for a few minutes and everyone will get out, sometimes with the exception of the people working on the tram. There is a constraint between the arrival and the departure of the tram. There is a constraint between the clock of the tram driver and the fact that the tram is departing. There is a constraint between the clock of the tram driver and the clock hanging in the kitchen. As such, the sociomaterial environment that we inhabit is replete with constraints. It might be next to impossible (and unnecessary) to figure out how these constraints come about, but it is the existence of these constraints that enable us to coordinate our behavior with respect to aspects of the environment to which we are not sensorily coupled. Just as the veterinarian is able to perceive what needs to be done to the dog’s leg while being sensorily coupled to the X-ray, we can perceive it is time to leave to take the tram by looking at a watch.

It is an open question how far back one can push the requirement for sensory coupling. Even if the tram were not yet actually present, we could still see the tram stop sign, we could still see the rails that lead it to, for example, the street called Ceintuurbaan in Amsterdam. In fact, nothing much is changed by the arrival of the tram because one can be familiar with this entire sociomaterial practice and attuned to the regularity of the arrival tram 3: another tram will arrive soon.

Even if the curtain is closed and we do not see the tram, then we can still see that we are in the familiar apartment, a place that is constantly placed next to the tram 3 stop. The clock in the kitchen might still inform us that we have to leave now to catch the next tram. Anyone with the right abilities and sensitivity to the regularities that allow one to reliably couple to the affordances will be able to coordinate with these distal aspects of the form of life in virtue of information about more local aspects, we suggest. So no direct sensorily coupling with an object (e.g. the tram) is necessary in order for an agent to be open to its affordances. Part of being skilled, of being at home in the situation, is exactly that of being able to adequately coordinate actions with respect to a great number of aspects of the environment in virtue of the presence of a small number of things that *reliably covary* with these aspects.

Needless to say, all of the constraints just mentioned are fallible, and none are lawful: the clock might be fast, the tram might be late, or the tram stop might be temporarily relocated due to construction work. Still, crucially, normally they provide the regularities that allow one to skillfully coordinate behavior in a form of life.

There are a few things to note here. First, this notion of regularity is not inconsistent with the Gibsonian account of lawful ecological information. Lawful ecological information, understood traditionally as a 1:1 specifying relationship between the structure of the light and the substances and surfaces of the environment, is a special case of the notion of general ecological information as we develop it here. When you grasp the cup of coffee in front of you, the structure of the light at some point *p* might fully specify the location and structure of the cup, such that it affords grasping. What we want to resist, however, is that affordances are limited *exclusively* to cases where we are lawfully coupled to an affordance.

For a skilled agent at home in her ecological niche, it is sufficient to be coupled to some relevant aspect of the sociomaterial practice even when that aspect is not present in the current environment. Even though we are not visually coupled to the beer inside the unopened beer can, the beer inside can still solicit us. Even with the curtains drawn, the tram can still afford catching due to the regularities in the form of life mentioned above.

Second, note that the account of regularities that we propose is an interesting case of niche construction: many of the constraints and regularities we have talked about in this section are products of human invention. The construction of clock time, especially the construction of time zones, led to a great explosion of things happening simultaneously or sequentially. A mobile phone can tell us when the tram arrives, even a tram in another city. It is an impressive technological feat that a pattern of light and sound at a point of observation somewhere else in a faraway country can appear in real time on a laptop in a Skype conversation. That is to say, it is not just the construction of material structures that change our ways of living, but also people actively inducing patterns that make events happen at regular moments. Within our framework, the regularities of time keeping induce new constraints between situations (cf. van Dijk and Withagen 2016). Without having to look out of the window, the light bouncing off the clock in the kitchen informs us that we should leave now in order to catch the tram and make it to the appointment on time.

Third, we want to be clear that the abilities that allow us to be informed by conventional constraints are not independent of lawful constraints. Indeed, the use of conventional constraints to couple to distal features of the world depends on the use of local lawful constraints. An apartment dweller can be coupled to the distant tram via the conventions connecting its arrival to the printed schedule, the way that the schedule constrains the actions of the conductor, and the conventions according to which she and the train conductor set their clocks. But she can only be coupled to the tram via these conventional constraints if she is also lawfully coupled to, for example, the clock in her kitchen via the lawful, optical constraints governing the light reflected off the hands of the clock’s hour and minute hands.

In this section, we have introduced the notions of *lawfulecological information *and *general ecological information.Lawful ecological information* pertains to the currently most influential Gibsonian notion, especially as used and developed by Turvey et al. (1981). It is defined as a lawful regularity in the ecological niche between structure at a point *p* in a medium and an aspect of the environment at point *q* such that there is a 1:1 specifying relationship between *p* and *q*. *General ecological information* is defined as any regularity in the ecological niche between different aspects of the environment (*x*and *y*) such that the occurrence of *x* makes *y* likely. The regularities in the ecological niche can be captured by the notion of *constraint*. Constraints are relationships such that something being in a particular state constrains the state in which something else can be. For example, an aluminum can having a beer logo constrains the possible contents of the can, or a shadow having a particular shape constrains the object from which the shadow originates.

The nature of these constraints can be the object of scientific research, but for the skilled agent it suffices to be sensitive to these constraints. Constraints might be brought about by complex causal structures (such as X-rays), by ecological laws (such as optics), conventions (driving on the right) or habits (beer in the fridge). In the human form of life all these constraints are meshed together. In the next Sect. 3, we will discuss how our notion of general ecological information can contribute to our understanding of episodes of “higher cognition”, but first we would like to deal with a potential objection to our account.

*Information and use*

One problem that emerges for a constraint-based notion of information, as proposed by us in this paper, has been articulated by Withagen and van der Kamp (2010). They argue that the extension of ecological information to include non-specifying constraints poses, among others, the problem that information cannot specify the object of perception. If a structure in the array covaries with multiple aspects of the environment, then the array itself cannot individuate the object of perception. In other words: why does the light structure of type fridge-presence specify beer-presence rather than, say, milk-presence, even though both are reliably present?

Withagen and Van der Kamp sketch two possible solutions to this problem. The first is to deny that information has to specify the perceptual object and to maintain that in fact the array carries information about all these aspects of the environment. Some internal process (such as an intention) then further determines which of these aspects is perceived. Withagen and Van der Kamp dismiss this option for they claim that a theory of information should, ideally, explain the object of perception and postulating internal process necessary to individuate the perceptual object violates the principles of ecological psychology.

An alternative solution, they hold, is to define information relationally and to deny that the array carries information independent of use (see also van Dijk et al. 2015). Following developmental systems theory (Oyama 1985/2000, 2000), they argue that information is not intrinsic to a structure (such as an optic array or a strand of DNA), but relative to a system for whom that structure makes a difference. As such, “the impact of sensory stimuli is a joint function of the stimuli and the sensing organism; the ‘effective stimulus’ is defined by the organism that is affected by it” (Oyama 1985/2000, p. 38).

We want to argue that although our particular constraint-based notion of information and the usage-based notion of information are substantially different, their resulting treatment of perception and action are not. First of all, Withagen and Van der Kamp make clear that they do not wish to deny “the highly structured energy patterns in the ambient arrays that animals *can* use” (p. 158, our italics). For reasons mentioned above, they just take issue with equating information with such patterns. Constraints are necessary, but not sufficient for information, according to Withagen and Van der Kamp. We take it that both the usage-based and our constraint-based approach to information agree that perception is the result of a system of constraints interacting with a perceptual system sensitive to only some of these constraints. Constraint-based theorists like us define one of the relata (the constraints) as information; usage-based theorists define the relation itself as information. Consequently, for the former information is cheap, ubiquitous and user-independent, while for the latter it is rare, special and user-relative.

We suggest then that as long as the different notions of information are kept apart, they are not incompatible. Information-as-constraint pertains to the regularities in the environment independent of use by a particular individual and *does not *specify either the object of perception or what is relevant. Information-as-use pertains to the relational significance of an environment for an agent and *does* specify the object of perception and what is relevant.

On our view, information and affordances are present in the ecological niche independent of the use by a particular agent. However, the animal cannot attune to all of these affordances at the same time, it needs to be *selectively *open or sensitive to only the relevant ones. While Withagen and Van der Kamp are worried that any notion of information that does not specify the object of perception requires some sort of “internal enrichment” (e.g. an intention) in order to arrive at the object of perception, we, instead, argue that it just takes a process of selective openness to arrive at only the relevant affordances, or solicitations.^9^

Despite these differences, the user-based and our constraint based approach agree, contrary to traditional ecological psychology, that perception and action should be understood as a function of the agent-environment system as a whole. To understand an animal’s directedness to its environment it is not sufficient to merely focus on the constraints and regularities in the form of life, but also to focus on how an agent is able to selectively be sensitive to or be invited by some affordances and not others. We have discussed in earlier work how to conceive of selective openness to affordances within radical embodied cognitive science and the Skilled Intentionality Framework (Chemero 2003a; Bruineberg and Rietveld 2014; Bruineberg et al. 2016).

## Information for engagement with affordances for “higher” cognition

In earlier work, we have discussed certain kinds of affordances for “higher” cognition (Rietveld and Kiverstein 2014; Rietveld and Brouwers 2017; Van Dijk and Rietveld 2017). For example, the possibility of judging correctly that this paper is written in English, or possibilities for imagining a future building. What is the nature of constraints and information involved in engaging with such an affordance? Based on the theory developed so far in this paper, this kind of affordance is to be understood in the same way as affordances for mundane activities like grasping a cup in the context of a particular situation.

How do we couple with what is absent in the sense of not immediately present to one of the senses? Notice that already in the very definition of an affordance there is an interesting notion of absence. Affordances are *possibilities* for action, and those actions must occur in the future. You *could* get your telephone out of your pocket now. Even when we are ready to act on an affordance, we are prepared for something that we *could* do, but what we could do is not yet done, so in a sense something is not yet there. There is no light bouncing off the future. This implies that there is something necessarily anticipatory or future-oriented in the perception of an affordance (Turvey 1992). This absence only increases when the affordances become more complex.

Crucially, we want to use affordances to talk about ‘higher order’ cognition in a way that views cognition as an unmediated contact of the skilled person with the regularities in the environment. The regularities apply at many different spatial and temporal scales, including at the scale of sociocultural practices. The individual’s skills (most of which are acquired via a process of education of attention in socio-cultural practices) provide access to the regularities of the world; some skills are primarily sensorimotor, such as grasping a cup, others are typically characterized as more abstract skills (e.g. imagination, but also appropriately grasping your own coffee cup rather than someone else’s from those on the table in front of you). Part of being skilled is knowing how to attune to the relevant pieces of information; i.e. coordinate with the relevant aspects of the environment.

*Imagination: general ecological information and anticipatory states of action readiness*

In this section, we will discuss some of the regularities involved in a specific case of imagination. Let us consider the real-life example of a team of architects designing an art installation, *The End of Sitting*, for the Chicago Architecture Biennial, while having previous built a similar installation in Amsterdam.^10^ The End of Sitting is a large architectural art installation in which visitors are invited to engage with a landscape of standing affordances. It is a rock-like structure that affords a variety of supported leaning, standing and hanging postures.^11^ Moreover, due to the large variety of positions offered and the temporary comfort offered by each of them, the installations affords switching positions about every 20–60 min (Rietveld et al. 2015; Withagen and Caljouw 2016; Caljouw et al. 2017; Rietveld 2016).

In the process of being responsive to the possibility of making the installation for Chicago, the architects are able to anticipate how the installation will be used because they have perceived how it is used in Amsterdam, both by observing visitors and by experiencing it themselves. The way people interact with the installation in Amsterdam is something very human: it is supported standing in different ways. Once one is familiar with how these human practices work in Amsterdam and that the human practices of supported standing in Chicago are sufficiently similar, one can imagine what will happen in the new installation in Chicago even though there are minor differences. But these differences are not radical: people are not leaning against air or against birds or earth worms in Chicago, they are leaning against materials that support leaning and they partake in the human form of life as well. The main differences are related to body posture: on average people are a bit less tall and a bit bigger in Chicago. That is to say, thanks to the similarities there will be a constraint between the practice in Amsterdam and the practice in Chicago, which enables the architects to be informed about the practice in Chicago by observing the practice in Amsterdam. Even though the architects in Amsterdam are not in sensory contact with Chicago in any standard sense, they are able to anticipate how their installation will be used in virtue of regularities concerning human bodies and practices of standing that hold both in Amsterdam and Chicago (plus minor adjustments for differences in body posture).

The architects, located in Amsterdam, are in touch with the practice of standing and leaning in Chicago, in a way similar to the thirsty person, situated in a socio-cultural setting in which beer is a common beverage, who perceives a closed beer can is in touch with the beer. In both cases the agent is coupled to a local aspect of its environment: a scale model in the studio and the fridge respectively, which, in virtue of constraints in the form of life bring the agent in touch with some distal aspect of the environment: the practice in Chicago and the beer in the fridge, respectively. This kind of imagination of supported standing in Chicago is not qualitatively different from tending towards a grip on the fridge, or the cup of coffee in front of me. The differences between the beer can and the case of imagination concern only the two relata of affordances: there are differences in the skills engaged and differences in the regularities of the environment involved. Note that these two relata (aspects of regular patterns in the sociomaterial environment on the one hand, and abilities on the other) are precisely the two relata of affordances (Rietveld and Kiverstein 2014). Ecological optics is just a special case of the class of regularities that can couple a skilled agent to its environment.

Imagination, as presented here, turns out to be a form of anticipation, made possible by skills and regularities of the environment. One consequence of this position is that, contrary to how ecological information is typically understood, general ecological information is not necessarily tied to a medium that grounds the informational relation. Whereas the informational relation between a point of observation and a beer can is grounded by the properties of the local medium, the informational relation between practices of supported standing in Chicago and in Amsterdam is not. Still, in the case of architectural practice, the information relation between a scale model and a point of observation is grounded in the local medium. The information connecting the scale model and the installation in Chicago is a feature of the sociomaterial practices of architects.

Imagination in architectural practices might seem to be a fairly specific form of imagination. Indeed, as Ryle (1949) famously noted, just as there is no canonical activity that is both necessary and sufficient for farming, there will be no single operation that is involved in all and only cases of imagination. Some forms of imagining (both in architecture and outside of it) might involve the active manipulation of aspects of the environment, some might be more like mind wandering, and some involve sitting still and imagining something by actively manipulating concepts or images (Gallagher 2017).

It is therefore an open question whether all cases of imagination can be understood in the same way. Take for example a science fiction writer writing about a completely imaginary universe. Even in such outlandish cases of science fiction, emphasis is put on a skill: the skill of *worldbuilding * (Wolf 2012). That is the art of imagining worlds in which some (combination of) physical, conventional or bodily constraints are different from one’s own environment. We hypothesize that such worldbuilding, just like the architectural case, is distributed over aspects of the environment, and, crucially, also requires a sensitivity to the interwovenness of constraints such as we developed in this paper. Although a full treatment of the practice of science fiction writing would require a research project of its own, including an understanding of the specific details, skills and contexts of such writing practices (and how to spell out all this in terms of skilled intentionality), we take it there is no reason to think imagination in such practices will be radically different from the architecture example provided in this section.

In sum, the example of the architects in Amsterdam anticipating how something will be used in Chicago shows that imagination involves dealing with locally absent users (i.e. distally present users) primarily on the basis of regularities in human behavior. Imagination is a form of anticipation made possible thanks to skills and the presence of regularities in our sociomaterial surroundings. Hence, there is no need to over-intellectualize imagination by understanding it in terms of mental representations.

Our basic claim here is that things in the form of life tend to happen in regular patterns that occur both locally and distally. Being attuned to these regularities allows the agent to let its behavior be guided by these patterns. Skills can be applied in new situations/instances appropriately because some of the regular patterns are the same across situations. Part of being skilled is knowing how to attune to the relevant pieces of general ecological information; i.e. connecting skills with the relevant aspects of the local and distal environment. When we are attuned to the regularities in the environment, we are able to be selectively open to those affordances that improve our grip on the situation, including affordances for activities that people have typically categorized as ‘higher’ cognition. What couples us selectively with the world is the action-readiness of the skilled individual to be sensitive to environmental constraints and act on relevant affordances.

## Conclusion

In this paper, we have presented an account of ecological information that is able to ground an animal’s skilled interactions with absent aspects of the environment. The other main contribution of this paper is to distinguish between *lawfulecological information* and *general ecological information*. While ecological psychology has typically focused on the former, emphasising lawful informational coupling between agent and environment, our more general notion of ecological information has broader applicability, especially when it comes to sociomaterial aspects of the environment and forms of so-called ‘higher’ cognition. We have defined *general ecological information* as any regularity in the ecological niche between different aspects of the environment (*x* and *y*) such that the occurrence of aspect *x* makes the occurrence of aspect *y* likely. The nature of this regularity can be captured by different kinds of constraints. Constraints can, among others, be resulting from complex causal structures (such as X-rays), laws at the ecological scale (such as optics), conventions (like driving on the right), or habits (such as having beer in the fridge). The existence of these regularities and constraints allows for the possibility of having the skill to be sensitive to these constraints; that is, these constraints allow situations to inform agents about what can be done. Lawful ecological information is best understood as a special case of general ecological information.

On our view, constraints make information available in our form of life, independently of any particular observer/agent. They pertain to the ways in which aspects of the environment tend to occur together, like an object and its shadow or an invitation and a party. General ecological information is understood as a relation between aspects of the (sociomaterial) environment (e.g. a house and its shadow), but it is not relative to a particular observer or agent. This does not contradict the fact that agents can generate certain kinds of information, for instance by moving.

We have discussed the Skilled Intentionality Framework (SIF). Skilled intentionality is coordinating with multiple affordances simultaneously. Affordances are relations between aspects of the (sociomaterial) environment and abilities available in a form of life. As such they are there also when no observer is locally present, but they are dependent on the existence of the form of life. Only when a kind of animal with the right leg-proportions and abilities arose, for example, did the log afford sitting on for that kind of animal. Solicitations, the relevant affordances in the concrete situation, are dependent on a particular individual’s state of action-readiness. So, whereas information and affordances are available in the environment of the form of life, solicitations are there relative to the concrete individual animal in the concrete situation.

What we have highlighted is the continuity between simple cases of engaging with affordances and behaviors that are typically categorized as ‘higher’ cognition, such as architectural design that deals with something that is currently absent but to be built in the future. In all these cases, it is the skillful attunement to the regularities that are there in the environment (lawful, conventional or sociomaterial) that allow the agent to be responsive to relevant affordances. The conception of affordances as relations between aspects of the sociomaterial environment and abilities available in a form of life allows us to circumvent artificial dichotomies between sensorimotor coordination and ‘representation-hungry problems’ (Clark and Toribio 1994) or the intelligibility of the interface between ‘contentless’ basic minds and enculturated and linguistic forms of cognition (Hutto and Myin 2013). Skilled agents are perfectly able to engage with and think about absent objects and sociomaterial practices, as long as they connect in regular ways with their environment.

However, one might wonder whether we have provided an ecological-enactive account of ‘higher’ cognition or have shown that what one might have thought of as ‘higher’ cognition is actually ‘lower’ cognition (i.e. only different in degree from animal cognition). Formally similar discussions occur in, for example, the field of artificial intelligence where a particular behavior (say, human-level competence in the game of Go) is a sign of genuine intelligent behavior until a computer can actually do it, after which it is understood as merely a case of pattern recognition.

We take it that ‘dealing with the absent’ and ‘dealing with sociocultural norms’ licenses the claim that in this paper we are ‘dealing with ‘higher’ cognition’. We consistently use scare quotes for ‘higher’ cognition exactly because our work shows that there might not be a hard demarcation between ‘lower’ and ‘higher’ cognition. What matters for understanding both forms of cognition is that they are forms of skilled action or, more precisely, skilled intentionality. Dealing with the absent shows up already in the anticipatory character of engagement with affordances and can be extended to cases like imagination and architectural design, situated in socio-cultural practices. Imagination is a form of anticipatory engagement with multiple affordances simultaneously, which is made possible by the presence of (and skilled familiarity with) regularities at different spatio-temporal scales in our sociomaterial surroundings. That is to say, imagination, like the other forms of ‘higher’ cognition discussed in this paper, is supported by general ecological information available in our human form of life. General ecological information does not only link the ambient array to structures or situations in the local environment, but also links between situations that are distant from one another in space and time.

## Footnotes

1 This also means that the broader understanding of affordances can do justice to (situated) normativity (Rietveld 2008; Chemero 2009) and the more restrictive (law-based) understanding cannot, which limits the extent in which the latter type of account is able to deal with many kinds of ‘higher’ cognition.

2 It is an open and interesting question to what extent these informational structures for ‘higher’ cognition figure in the form of life of other animals as well. A comparative study is beyond the scope of this paper.

3 In the following, the pronoun “we” will be used for any work authored by at least one of the authors of this paper.

4 As we will discuss below, this does not deny the fact that local lawful constraints can enable the *use* of conventional constraints by a particular individual.

5 Since this paper only deals with ecological information and not with other kinds of information, we will in the following use information and ecological information interchangeably.

6 Recall that it is skills that give an individual intentional access to the world (see Sect. 1).

7 Some authors (van Dijk et al. 2015) state that “information about” implies normative evaluability (i.e. true or false, accurate or inaccurate). They therefore claim that conceiving of information independent of, and prior to, use is inappropriate. See section “Information and use” for a discussion of their claim.

8 One example Runeson discusses is the Ames-room (Wittreich 1952), a spatially distorted room which gets “interpreted” as a rectangular room, giving rise to size illusions. Runeson argues that although alternative configurations are geometrically possible, they do violate conventional and pragmatic constraints such as that rooms have rectangular and horizontal floors and vertical walls.

9 The problem for the user-based account is to explain how the agent is selectively sensitive to some constraints and not to others, or, in their own words, how an agent modulates the invitational character, or tunes the coupling strength (Withagen et al. 2017). of some affordances and not of others without presupposing some sort of internal agent or mysterious process that does this tuning.

10 This section discusses one kind of imagination as used by architects in a design process. There might be other forms of imagination, such as for example daydreaming, that require a slightly different analysis.

11 Many human activities can be performed skillfully exactly because they rely on carefully designed niches. However, the design process of a new aspect of a niche itself (for example, a landscape of affordances for supported standing) is neither a blind process, nor a process in which one of the architects has the end result in mind and merely realizes it in the world (Ingold 2013). Rather, the construction of a new aspect of a niche is itself a result of skilled practices enabled by the skills of the architects and the affordance provided by the architecture studio (including for example the possibilities to build cardboard models and to make 3D-drawings) (Rietveld et al. 2015; Rietveld and Brouwers 2017).

## References

[CR1] Barwise J, Perry J (1981). Situations and attitudes. Journal of Philosophy.

[CR2] Barwise J, Perry J (1983). Situations and attitudes.

[CR3] Bruineberg Jelle, Kiverstein Julian, Rietveld Erik (2016). The anticipating brain is not a scientist: the free-energy principle from an ecological-enactive perspective. Synthese.

[CR4] Bruineberg J, Rietveld E (2014). Self-organization, free energy minimization, and optimal grip on a field of affordances. Frontiers in Human Neuroscience.

[CR5] Byrge L, Sporns O, Smith LB (2014). Developmental process emerges from extended brain–body-behavior networks. Trends in Cognitive Sciences.

[CR6] Caljouw SR, de Vries R, Withagen R (2017). RAAAF’s office landscape The End of Sitting: Energy expenditure and temporary comfort when working in non-sitting postures. PLoS One.

[CR7] Chemero A (2003). An outline of a theory of affordances. Ecological Psychology.

[CR8] Chemero A (2003). Information for perception and information processing. Minds and Machines.

[CR9] Chemero A (2009). Radical embodied cognitive science.

[CR10] Chiel HJ, Beer RD (1997). The brain has a body: Adaptive behavior emerges from interactions of nervous system, body and environment. Trends in Neurosciences.

[CR11] Clark A, Toribio J (1994). Doing without representing?. Synthese.

[CR12] Costall A (1999). An iconoclast’s triptych: Edward Reed’s ecological philosophy. Theory & Psychology.

[CR13] Dreyfus H, Kelly SD (2007). Heterophenomenology: Heavy-handed sleight-of-hand. Phenomenology and the Cognitive Sciences.

[CR14] Frijda N (2007). The laws of emotion.

[CR15] Gallagher S (2017). Enactivist interventions: Rethinking the mind.

[CR16] Gibson, J. J. (1979/2015). *The ecological approach to visual perception*. Boston, MA: Houghton Mifflin.

[CR17] Golonka S (2015). Laws and Conventions in Language-Related Behaviors. Ecological Psychology.

[CR18] Golonka, S., & Wilson, A. D. (2016). Ecological Representations. *bioRxiv.*10.1101/058925.

[CR19] Heft H (2001). Ecological psychology in context: James Gibson, Roger Barker, and the legacy of William James’s radical empiricism.

[CR20] Hutto DD, Myin E (2013). Radicalizing enactivism: Basic minds without content.

[CR21] Ingold T (2000). The perception of the environment: Essays on livelihood, dwelling and skill.

[CR22] Ingold T (2011). Being alive: Essays on movement.

[CR23] Ingold T (2013). Making: Anthropology, archaeology, art and architecture.

[CR24] Jonas, H. (1966/2001). *The phenomenon of life: Toward a philosophical biology*. Evanston, IL: Northwestern University Press.

[CR25] Lee DN, Reddish PE (1981). Plummeting gannets: A paradigm of ecological optics. Nature.

[CR26] Merleau-Ponty, M. (1945/2002). *Phenomenology of perception.* London: Routledge.

[CR27] Merleau-Ponty M (2003). Nature: Course notes from the Collège de France.

[CR28] Noë A (2012). Varieties of presence.

[CR29] Oyama, S. (1985/2000). *The ontogeny of information: Developmental systems and evolution*. New York: Cambridge University Press.

[CR30] Oyama S (2000). Evolution’s eye: A systems view of the biology-culture divide.

[CR31] Reed ES (1996). Encountering the world: Toward an ecological psychology.

[CR32] Rietveld E (2008). Situated normativity: The normative aspect of embodied cognition in unreflective action. Mind.

[CR33] Rietveld E, Paglieri F (2012). Bodily intentionality and social affordances in context. Consciousness in interaction: The role of the natural and social context in shaping consciousness.

[CR34] Rietveld E (2016). Situating the embodied mind in a landscape of standing affordances for living without chairs: Materializing a philosophical worldview. Sports Medicine.

[CR35] Rietveld E, Brouwers AA (2017). Optimal grip on aordances in architectural design practices: An ethnography. Phenomenology and the Cognitive Sciences.

[CR36] Rietveld, E., Denys, D., & Van Westen, M. (In press). Ecological-enactive cognition as engaging with a field of relevant affordances: The skilled intentionality framework (SIF). In A. Newen, L. de Bruin, & S. Gallagher (Eds.), *Oxford handbook of 4E cognition*. Oxford University Press.

[CR37] Rietveld E, Kiverstein J (2014). A rich landscape of affordances. Ecological Psychology.

[CR38] Rietveld E, Rietveld R, Mackic A (2015). The end of sitting: Towards a landscape of standing affordances. Harvard Design Magazine.

[CR39] Runeson S (1988). The distorted room illusion, equivalent configurations, and the specificity of static optic arrays. Journal of Experimental Psychology: Human Perception and Performance.

[CR40] Runeson S (1989). A note on the utility of ecologically incomplete invariants. International Society for Ecological Psychology Newsletter.

[CR41] Runeson S, Frykholm G (1983). Kinematic specification of dynamics as an informational basis for person and action perception: Expectation, gender recognition, and deceptive intention. Journal of Experimental Psychology: General.

[CR42] Ryle G (1949). The concept of mind.

[CR43] Sedgewick, H. (1973). *The visible horizon*, Unpublished Doctoral Dissertation, Cornell University.

[CR44] Stoffregen TA (2003). Affordances as properties of the animal-environment system. Ecological Psychology.

[CR45] Thompson E (2007). Mind in life: Biology, phenomenology, and the sciences of mind.

[CR46] Turvey M (1992). Affordances and prospective control: An outline of the ontology. Ecological Psychology.

[CR47] Turvey MT, Shaw RE, Reed ED, Mace WM (1981). Ecological laws of per- ceiving and acting: In reply to Fodor and Pylyshyn. Cognition.

[CR48] Von Uexküll, J. (1934/1992). A stroll through the worlds of animals and men: A picture book of invisible worlds. *Semiotica *89(4): 319–391 (originally published in 1934).

[CR49] Van Dijk, L. & Rietveld, E. (2017) Foregrounding sociomaterial practice in our understanding of affordances: The Skilled Intentionality Framework. *Frontiers in Psychology.*10.3389/fpsyg.2016.01969 10.3389/fpsyg.2016.01969 PMC5220071 28119638

[CR50] van Dijk L, Withagen R (2016). Temporalizing agency: Moving beyond on-and offline cognition. Theory & Psychology.

[CR51] van Dijk L, Withagen R, Bongers RM (2015). Information without content: A Gibsonian reply to enactivists’ worries. Cognition.

[CR52] Withagen R, Araújo D, de Poel HJ (2017). Inviting affordances and agency. New Ideas in Psychology.

[CR53] Withagen R, Caljouw SR (2016). “The end of sitting”: An empirical study on working in an office of the future. Sports Medicine.

[CR54] Withagen R, de Poel HJ, Araújo D, Pepping GJ (2012). Affordances can invite behavior: Reconsidering the relationship between affordances and agency. New Ideas in Psychology.

[CR55] Withagen R, van der Kamp J (2010). Towards a new ecological conception of perceptual information: Lessons from a developmental systems perspective. Human Movement Science.

[CR56] Wittgenstein L (1953). Philosophical investigations.

[CR57] Wittgenstein L, Klagge JC, Nordmann A (1993). Cause and effect: Intuitive awareness. Philosophical occasions 1912–1951.

[CR58] Wittreich WJ (1952). The Honi phenomenon: A case of selective perceptual distortion. The Journal of Abnormal and Social Psychology.

[CR59] Wolf MJ (2012). Building imaginary worlds: The history and theory of subcreation.

